# The Effects of the Exogenous Melatonin on Shift Work Sleep Disorder in Health Personnel: A Systematic Review

**DOI:** 10.3390/ijerph191610199

**Published:** 2022-08-17

**Authors:** Bárbara Carriedo-Diez, Javier Lucas Tosoratto-Venturi, Carmen Cantón-Manzano, Carmina Wanden-Berghe, Javier Sanz-Valero

**Affiliations:** 1 Manacor Hospital, Occupational Health Service, 07500 Manacor, Spain; 2 Son Llàtzer Hospital, Occupational Health Service, 07198 Palma, Spain; 3 Getafe Hospital, Occupational Risk Prevention Service, 28905 Getafe, Spain; 4 Foundation for the Promotion of Health and Biomedical Research in the Valencian Region (FISABIO), Health and Biomedical Research Institute of Alicante (ISABIAL), 03010 Alicante, Spain; 5 Department of Public Health and History of Science, Miguel Hernández University, 03550 Alicante, Spain; 6 Carlos III Health Institute, National School of Occupational Medicine, 37311 Madrid, Spain

**Keywords:** health personnel, melatonin, sleep disorders, circadian rhythm, occupational health

## Abstract

(1) Background: To know the medical documentation related to exogenous melatonin in sleep disorders caused by shift work in health personnel; (2) Methods: Systematic and critical review. Data were obtained by looking up the bibliographic data base: MEDLINE (via Pubmed), Embase, Cochrane Library, Scopus, Web of Science, Latin American and Caribbean literature in Health Sciences (LILACS) and Medicine in Spanish (MEDES). The used terms, as descriptors and text in the title and abstract record fields, were “Health Personnel”, “Melatonin” and “Sleep Disorders”, Circadian Rhythm, by using the following filters: “Humans”, “Adult: 19+ years” and “Clinical Trial”. The search update was in December 2021. The documentary quality of the articles was assessed using the CONSORT questionnaire. (3) Results: Having applied the inclusion and exclusion criteria, 10 clinical essays were selected out of 98 retrieved references. CONSORT scores ranged from a minimum of 6.0 to a maximum of 13. 7 with a median of 10.2. According to the SIGN criteria, this review presented “1-“evidence with a grade of recommendation B. The intervention dose via administration of exogenous melatonin ranged between 1 and 10 mg. It was not mentioned whether the route of administration was by fast or slow absorption. The outcomes showed decreased daytime sleepiness, lessened sleep onset latency, diminished night-time awakenings, increased total sleep period and improved daytime attention in the melatonin-treated group; (4) Conclusions: Exogenously administered melatonin is effective in shift worker health personnel that are suffering from sleep disorders, and given its low adverse effects and tolerability, it might be recommended. A great disparity was evidenced in terms of dose, follow-up periods and type of melatonin, small participant population, same age ranges and young age. Therefore, new trials would be needed to amend these observations in order to have full evidence that is able to ensure the efficacy of exogenous melatonin in the studied population.

## 1. Introduction

In modern society, the economy operates 24/7 (24 h a day, 7 days a week) with supply and demand principles, constantly. Thus, shift work is a common experience for many workers in the contemporary world. In simplified terms, shift work has been defined as “a way of organizing daily working hours in which different people or teams work in succession to cover the full 24 h” [1].

Consequently, a considerable part of employees is compelled to work outside regular daytime hours, so that approximately one out of every five European employees is exposed to schedules that include night shifts. This night work schedule may lead to natural circadian rhythm disorders in the workers, as well as altering their biological functions, which subsequently might affect their health and welfare [2].

Health care personnel are among of the professional groups that have shift work as a regular practice, including night-time shifts, and it has already been proven that this work rhythm is related to circadian rhythm disorders. Insufficient sleep is associated with deteriorated daytime functioning, physical health problems, anxiety, depression, fatigue and increased cardiovascular risk.

The pharmacological interventions may relieve sleepiness in shift workers and improve alertness during shift work or reduce sleep disorders outside the work [3]. Thus, in an attempt to regulate the circadian rhythm and sleep, several lines of research opted for melatonin as a therapy indicated for diseases and sleep disorders in humans [4]. 

Herxheimer et al. [5] revised different studies tied to jet lag concluding that melatonin was effective in forestalling or lessening jet lag, and its occasional short-term use appeared to be safe. Nevertheless, in this systematic review, the effects of melatonin have not yet been proven the in shift workers and its possible side effects in a long-term period. 

In 2014, Liira et al. [6] assessed the effectiveness of pharmacological interventions to lessen sleepiness or improve alertness at work and reduce sleep disorders outside working hours. However, they did not focus on melatonin exclusively, nor they target health care professionals. On the other hand, they draw the conclusion that there was a need for better-quality studies on the effectiveness of all pharmacological agents that induce sleep or promote alertness in shift workers. 

Today, it is well-known that melatonin is the main hormone involved in the regulation of oscillation between sleep and wakefulness. It is easily synthesized and can be administered orally, which has led to interest in its use in the treatment of one of the most prevalent human pathologies, insomnia. Nevertheless, despite the undeniable theoretical appeal of this approach to the problem of insomnia, the scientific evidence supporting the possible benefit of this replacement therapy is scarce. Not even the dosages, recommendations on who should receive it or the most appropriate pharmacological formulation are well-defined [7]. 

Therefore, the aim of the present study was to review and critically analyze the effects of exogenous melatonin on sleep disorder caused by shift work in health personnel.

## 2. Materials and Methods

### 2.1. Design

Critical analysis of the retrieved works through systematic technique.

The structure of this review followed the Preferred Reporting Items for Systematic Reviews and Meta-Analyses (PRISMA) [8] checklist for systematic reviews.

### 2.2. Data Souces

Data were procured from direct consultation and access, via the Internet, to the following health sciences bibliographic databases: MEDLINE (via PubMed), Embase, Cochrane Library, Scopus, Web of Science, Latin American and Caribbean Health Sciences Literature (LILACS).

### 2.3. Information Processing

To define the search terms, the Thesaurus of Health Sciences Descriptors (DeCS) developed by the Latin American and Caribbean Center for Medical Sciences Information (BIREME) and its equivalence with the Medical Subject Headings (MeSH) established by the U.S. National Library of Medicine were consulted. 

From the study of the hierarchical structure of both the Thesaurus and their indexing cards, the following equations were appropriate:

- Population: Health personnel—persons who work in the provision of health services, either as individual practitioners or as employees of health institutions and programs, even if they do not have professional training, and whether or not they are subject to public regulation.
  “Health Personnel”[Mesh] OR “Health Personnel”[Title/Abstract] OR “Health Care Provider*”[Title/Abstract] OR “Healthcare Provider*”[Title/Abstract] OR “Healthcare Worker*”[Title/Abstract] OR “Health Care Professional*”[Title/Abstract] OR “Nurse*”[Title/Abstract] OR “Pharmacist*”[Title/Abstract] OR “Physician*”[Title/Abstract] OR “Health Care Personnel”[Title/Abstract] OR “Health Care Practitioner*”[Title/Abstract] OR “Health Care Worker*”[Title/Abstract] OR “Health Profession Personnel”[Title/Abstract] OR “Healthcare Personnel”[Title/Abstract] OR “Healthcare Practitioner*”[Title/Abstract] OR “Healthcare Professional*”[Title/Abstract]
- Intervention: Melatonin—a biogenic amine found in animals and plants; in mammals, melatonin is produced by the pineal gland. Melatonin secretion increases in darkness and decreases during light exposure. Melatonin is involved in the regulation of sleep, mood and reproduction. Melatonin is also an effective antioxidant.
  “Melatonin”[Mesh] OR “Melatonin”[Title/Abstract] OR “Melatonina”[Title/Abstract]
- Outcome: Sleep disorders, circadian rhythm–dyssomnias associated with disruption of the normal 24-h sleep wake cycle secondary to travel shift work, or other causes.
  “Sleep Disorders, Circadian Rhythm”[Mesh] OR “Sleep Wake Schedule Disorder*”[Title/Abstract] OR “Circadian Rhythm Sleep Disorder*”[Title/Abstract] OR “Disturbed Nyctohemeral Rhythm*”[Title/Abstract] OR “Sleep Wake Cycle Disorder*”[Title/Abstract] OR “Shift Work Sleep Disorder*”[Title/Abstract] OR “Non 24 Hour Sleep Wake Disorder*”[Title/Abstract] OR “Nonorganic Sleep Wake Cycle Disorder*”[Title/Abstract] OR “Advanced Sleep Phase Syndrome*”[Title/Abstract] OR “Delayed Sleep Phase Syndrome*”[Title/Abstract] OR “Circadian Rhythm Sleep-Wake Disorder*”[Title/Abstract] OR “Sleep Phase Disorder*”[Title/Abstract] OR “Sleep Phase Syndrome*”[Title/Abstract] OR “Sleep Wake Phase Disorder*”[Title/Abstract] OR “Sleep Wake Phase Syndrome*”[Title/Abstract] OR “Sleep Wake Schedule Disorder*”[Title/Abstract]

The final search equation was developed for use in the MEDLINE database, via PubMed, through the Boolean union of the three suggested equations: Population AND Intervention AND Outcome (following the PIO format), using the filters: “Humans” and adults “Adult: 19+ years”.

This strategy was subsequently adapted to the characteristics of each of the other consulted databases, performing the search from the first available date in each of the selected databases until December 2021. In addition, a supplementary search was carried out to lessen the possibility of publication bias by manually searching the reference lists of the articles that were selected for the review. Moreover, the list of similar articles provided by MEDLINE was revised in each of the selected trials. Furthermore, experts were contacted to ascertain the possible existence of grey literature (materials and research developed by organizations unaffiliated with traditional academic or commercial publications that are disseminated through other distribution channels).

### 2.4. Final Selection of Articles

Articles that fulfilled the following criteria were selected for review and critical analysis:

- Inclusion: adjust the objectives of the search as follows: there is a treatment with exogenous melatonin in a clinical trial published in peer-reviewed journals and written in English, Spanish, Portuguese, Italian or German.
- Exclusion: The main problem in those articles where the full text could not be found lies in the fact there was no association between intervention and the outcome under study (criterion of causality); other articles included a non-adult population (under 18 years of age).

The selection of the corresponding articles was performed by the authors of this review. To validate the inclusion of the articles, the assessment of the selection concordance (kappa index) was set to be greater than 0.60 [9]. Provided that this condition is fulfilled, possible discrepancies were resolved by consensus among all the authors of this review.

### 2.5. Documentary Quality, Level of Evidence, Degree of Recommendation and Study of Biases

The adequacy of the selected articles was assessed using the CONSORT (CONsolidated Standards of Reporting Trials) guidelines for reporting observational studies [10], which contains a list of 25 essential checkpoints to be described during the publication of these papers. For each selected article, one point was assigned for each present item (if not applicable, not scored). When an item was made up of several sections, these were assessed independently, giving the same value to each of them and then averaging them as the result for that item. In no case was the total score of one point per item exceeded. 

The recommendations and grades of the Scottish intercollegiate Guidelines Network Grading Review Groups (SIGN) [11] were used to determine the level of evidence. The quality of the evidence was assessed using the GRADE System (Grading of Recommendations, Assessment, Development and Evaluation) [12].

The tool modified by the Cochrane Collaboration [13] was utilized to assess the possible biases of the trials included in the review: bias was assessed as a judgement (yes, no or unclear) for the dimensions selection, performance, outcome detection, attrition, reporting and other. 

The RoB.2 tool was used to assess the risk of methodological bias in the reviewed articles [14].

### 2.6. Data Extraction

The control of data amendment was performed by double tables that allowed the detection of divergences and their correction by re-consulting the originals. 

The multiplatform program ZOTERO (bibliographic reference manager developed by the Center for History and New Media at George Mason University) was used for the refinement of duplicate records (present in more than one database). 

The Burton–Kebler half-period (BK) and the Price index (PI) were calculated to determine the timeliness of the studies. 

To systematize and promote the understanding of the outcomes, articles were classified according to the variables under study considering the following data: first author, year of publication, studied population, country and period of the study, conducted intervention and main result motivated by the effect of the action. 

### 2.7. Data Analysis

Data related to information retrieval were presented in terms of frequency and percentage.

To determine the BK, the median age was calculated regarding the time range analysed, and the PI was computed by the percentage of articles with an age of less than 5 years. 

The measure concordance was performed using IK to ascertain the adequacy of the selection of articles. The relationship between authors was considered well-founded when its value was greater than 60% (good or very good concordance strength). 

The CONSORT questionnaire scores were analysed using the median, maximum and minimum scores. The evolution of these grades over the years of publication was procured using Pearson’s correlation analysis.

### 2.8. Ethical Aspects

All data were obtained from the accepted articles for review. Thus, in accordance with Law 14/2007 on biomedical research [15], the approval of the ethics committee was not required when using secondary data.

## 3. Results

Having applied the search criteria, a total of 98 references were retrieved: 28 (28.57%) in MEDLINE (via PubMed), 0 (0%) in Embase, 6 (6.12%) in Cochrane Library, 59 (60.20%) in Scopus, 5 (5.10%) in Web of Science and 0 (0%) in LILACS. No papers were retrieved from the MEDES bibliographic database. Consultation of the bibliographic lists of the selected articles resulted in the selection of nine studies.

**Figure 1:**
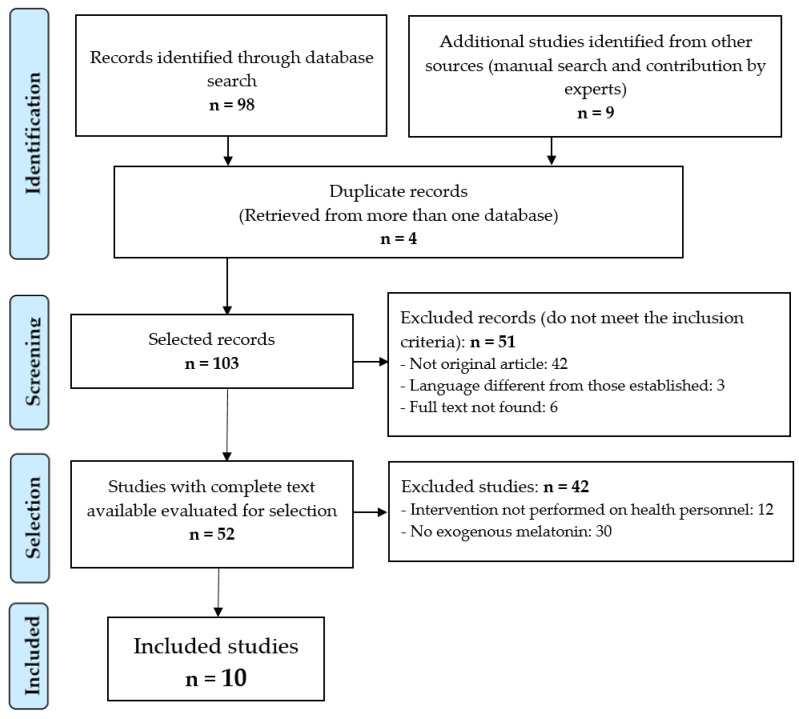
Identification and selection of the studies.

After filtering out four repeated records and applying the inclusion and exclusion criteria (Figure 1), it was possible to select 10 papers [16,17,18,19,20,21,22,23,24,25] for review and critical analysis; see Table 1.

Agreement on the pertinence of the selected studies amid the reviewers, calculated using the kappa index, was 74.13% (*p* < 0.01).

The selected articles had an obsolescence according to the Burton–Keber Index equal to 17.50 years, with a price index of 7.14%. By volume, 1998 had the highest number of published papers, from which three articles were chosen for the review [23,24,25].

When assessing the adequacy of the studies, using the CONSORT verification guide, the percentages of compliance ranged from a minimum of 6.02% to a maximum of 13.71%, with an average of 10.20. A moderate direct linear trend was observed, although it was not significant (R^2^ = 0.39, *p* = 0.055); see Table 2.

Based on the SIGN criteria, this review presented evidence with a grade of 1, systematic review of randomized clinical trials or randomized clinical trials with a high risk of bias, with a grade of B (a body of evidence that includes studies applied directly to the target population and showing overall consistency of results or extrapolation from studies rated as 1). The assessment of the evidence using the GRADE system demonstrated that this review would present a moderate level of evidence.

**Figure 2:**
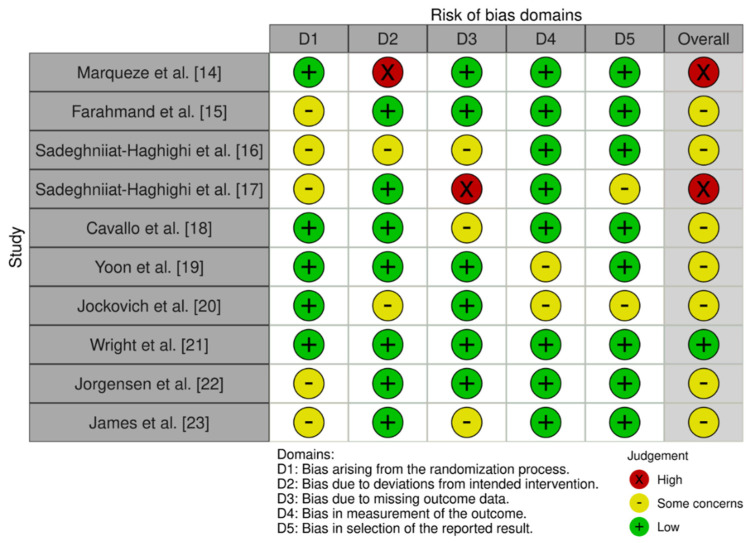
Methodological risk assessment of the clinical trials using RoB 2 tool.

The study of the trial’s biases encompassed in the review can be consulted in Table 3. The assessment conducted with the RoB. 2 tool for the methodological risk bias can be consulted in Figure 2.

According to the selection criteria, all reviewed studies were controlled randomised clinical trials. By the same token, all of them presented a crossover design except the of Cavallo et al. [20].

The United States was the country that contributed the largest number of trials, with 5 papers [20,22,23,24,25], followed by Iran presented three studies [17,18,19]. No trials with European affiliation were selected.

The majority population in the reviewed trials were resident physicians in 4 out of the 10 papers [17,20,22,24], followed by nursing staff in 3 other articles [16,19,21]. Another studied population was shift workers with difficulty falling asleep [18] (no further detail), generally physicians [23] and paramedics on night shifts [25].

The trial with the largest number of individuals included was that of Sadeghniiat-Haghighi et al. [19], with n = 86, whereas the trial with the smallest population belonged to Yoon et al. [21], with n = 12. The male/female ratio was mostly female, except for the studies performed by Farahmand et al. [17] and Jockovich et al. [22], although in the 2016 paper conducted by Sadeghniiat-Haghighi et al. [18], this ratio was not included.

The ages ranged between 20 years [25] and 46 years [19] with a median of 31.21 years.

The intervention period ranged from 1 to 5 days. The paper by Jorgensen et al. [24] was the only one with more than one period, grouping overnight shifts between 2 and 5 days.

In the crossover trials, the washout period between exogenous melatonin and placebo and placebo administration was a maximum of 28 days: consecutive days in the work of James et al. [25], 4 days in the case of the 2008 paper by Sadeghniiat-Haghighi et al. [19] and 15 days in reference to the 2016 trial conducted by Sadeghniiat-Haghighi et al. [18].

### 3.1. Performed Interventions

Reasonably, the intervention in the reviewed trials was based on the administration of exogenous melatonin, which was ingested in all cases after the night shift and between 30 and 60 min before bedtime.

Doses varied in a rather heterogeneous range from 1 to 10 mg: 4 trials utilize 3 mg melatonin [16,17,18,20]; 2 studies administer 5 mg [19,24]; another use 6 mg [21]; 1 article uses 10 mg [24] and another paper utilizes 1 mg of melatonin [22].

In the reviewed studies, no mention was found regarding the use of fast or slow absorbing exogenous melatonin.

It is noteworthy that none of the studies reported measuring endogenous melatonin level before starting the intervention, nor was it reported that investigators monitored the participants’ diets. It was only the trial carried out by Jorgensen et al. [24] that excluded two individuals from the study for drinking alcohol or taking sedatives, and they had also been mandated to limit caffeine intake. By the same token, James et al. [25] called for limitations on alcohol and caffeine consumption. 

Yoon et al. [21] and Jockovich et al. [22] requested that no alcohol should be drunk before the study.

### 3.2. Results of the Interventions

There was a significant lessening (*p* < 0.001) in daytime sleepiness after taking melatonin compared with the placebo group in the study conducted by Marqueze et al. [16] and from the second night onwards in the study performed by Farahmand et al. [17] (*p* < 0.003).

A meaningful reduction (*p* < 0.05) of sleep onset latency in the intervention group was demonstrated in the 2008 and 2016 studies carried out by Sadeghniiat-Haghighi et al. [18,19].

In the paper by James et al. [25], a significant decrease (*p* < 0.05) in nocturnal awakenings was observed in the case group.

In the trials of Yoon et al. [21] and Jorgensen et al. [24], there were increases (*p* < 0.05) in total night-time sleep period. 

Cavallo et al. [20] found an increase (*p* = 0.03) in daytime alertness in the melatonin-treated group.

## 4. Discussion

Having followed the recommendations on the objectives of a systematic review [26], this current review synthesized the pertinent information related to the effects of exogenous melatonin on sleep disorder caused by shift work in health personnel providing the scientific community with evidence that may promote new interventions for the protection of employees. Furthermore, this study is classified within the World Health Organisation’s strategy, which stresses the importance of establishing primary prevention and interventions aimed at improving occupational health [27].

The timeliness, or rather obsolescence, of the reviewed articles was higher than expected for the area of knowledge; however, it was akin to the previous occupational health reviews. This assertion is reflected in the fact that most of the retrieved studies were published before 10 years ago, which highlights the need for updating [28,29].

According to the assessment performed through the CONSORT criteria, the evaluation of the level of evidence and recommendation of the studies included in this review was to a certain extent lower than that observed in current systematic reviews on occupational health [30,31]. The study of the non-significant time trend in document adequacy contradicted the expected evolution of increased fulfilment in the course of time. Notwithstanding this, the non-compliance of older articles with the quality guidelines was to be expected as the first papers using the CONSORT criteria dated back to 1996 [32] and their use was progressive. In addition, Turner et al. [33] showed that the adoption of these criteria has resulted in improvements in the quality of the articles.

If the clinical trials have inadequate methodology, especially if the final description of the trial does not contain certain information, these issues are a hurdle for readers since they are unable to judge adequately the validity of the study and the scientific evidence related to the results will be very limited [34].

It is worth noting that most of the selected studies did not specify all the measures taken to address the potential sources of bias, nor did they describe the reasons for the loss of participants at each stage of the research or perform further analyses of interactions or sensitivity. This is why they did not earn higher scores.

The SIGN criteria ascertained that the level of evidence and the grade of recommendation of this study were consistent and akin to that observed in previous studies. Although some studies were subject to more bias than others and therefore supported the conclusions weakly [35]. The conclusions of many occupational health studies are still not based on the higher possible evidence [36]. This may be due to the experimental designs of the primary studies, including clinical trials, which are considered robust but may not be suitable for assessing occupational health interventions as they generally have long-term effects. Furthermore, in this review, the interventions were not measured specifically in the field of workers’ health. 

Similarly, the assessment of the evidence using the GRADE System demonstrated that the present review would present a moderate level of evidence, in other words, moderate confidence in the estimate of the effect with the possibility that the current effect was distant from the observed one. Nevertheless, the subsequent studies may have a significant impact on the monitorization confidence [12].

Using the tool modified by the Cochrane Collaboration [13] for the assessment of the potential biases of the trials included in the review confirmed what was observed with the RoB. 2 tool. 

The predominance of USA affiliation is a well-known fact and is widely reported in the scientific literature thanks to the power of its universities and the significant public and private funding of its institutions and research centres, which make positive contributions [37]. The anglophone affiliation of most of the articles and the fact that they were mainly written in English was expected, since this language is the chosen one for the publications of most of the articles, as doing so in a different language is negative for visibility. Additionally, the number of anglophone journals found in the main bibliographic databases is higher, and publishing in them facilitates citation [38,39].

The recruited health care personnel in the trials (resident doctors and nurses) may suggest that these groups are the ones who mostly work emergency shifts. On this point, Deschamps Perdomo et al. [40] demonstrated that nursing was one of the health sectors most affected by shift work, a situation also pointed out in the work of Vásquez-Trespalacios et al. [41]. On the other hand, it is noteworthy that the other health care group was resident doctors, albeit without considering the shift patterns in this not yet fully trained professional group working at night may aftermath both patient safety and the health of the professional, since it increases the risk of making bad decisions or even mistakes [42]. In short, there were few studies that might include a more general health care population.

The fact that the population was predominantly female meet the requirement of the World Health Organisation’s document “Gender equity in the health workforce: analysis of 104 countries” [43], which states that women constitute 70% of health and social sector workers.

The population size, collected in the reviewed trials, was considered small, and according to Vásquez-Trespalacios et al. [41], there were no sufficient studies with sample sizes that can establish adequate melatonin levels in night shift workers.

The fairly young age range was consistent with the studied health care personnel, especially when considering that most of the trials had been conducted on resident doctors. Melatonin is the main hormone involved in the regulation of the fluctuation amidst sleep and wakefulness, and its production reduces with age in an inversely proportional relationship to the frequency of poor sleep quality, which underpinned the argument that the deficit of melatonin is partially responsible for these disorders [7]. Therefore, despite of the irrefutable theoretical appeal of this approach to the problem of insomnia, the scant scientific evidence supporting the possible benefit of this substitution has not been reviewed since it does not include populations over 50 years of age. Melatonin as a drug is recommended in adults ≥ 55 years with primary insomnia and for a maximum of 13 weeks. The European Medicines Agency reports a small effect in a reduced group of adult/elderly population [44].

The follow-up period was too short for assessing the results of the intervention, a period of several weeks, even months, is considered necessary to assess the outcomes [37,45]. On the one hand, it was evinced that melatonin is not effective in the treatment of most primary sleep disorders with short-term use (4 weeks or less). On the other hand, it was proved that melatonin is effective in the treatment of delayed sleep phase syndrome with short-term use [46].

In the performed interventions, it was possible to verify the lack of discrimination between the fast and slow absorption of exogenous melatonin. It should be considered that melatonin has linear kinetics. It is rapidly absorbed and reaches peak dose in about 40 min [7], so the administration times observed (between 30 and 60 min before bedtime) would be correct. Nonetheless, its biological availability is low because of a significant hepatic first-pass effect (more than 90% of circulating melatonin is metabolized in the liver). In accordance with these pharmacokinetic characteristics, immediate-release melatonin would be more suitable for sleep induction, while prolonged-release melatonin would be more effective for sleep maintenance. In addition, this latter characteristic of melatonin seems to better simulate physiological levels observed naturally during the night and is probably more effective in improving sleep disorder.

Thus, in sleep initiation disorder, the administration of fast-acting melatonin is recommended [47], and in mixed and maintenance sleep disorder as well as in cases of early awakening, prolonged-release melatonin is preferable [48]. On account of this, it should have been indicated which type of melatonin had been administered in the reviewed trials.

The doses collected in the trials were quite heterogeneous and for the adult population were not considered high. The standard doses used in the studies ranged from 1 to 10 mg, although there is currently no definitive “best” dose. Doses in the 30 mg range are considered to be harmful.

Nevertheless, a previous Cochrane review [5] affirmed that daily doses of melatonin of 0.5 and 5 mg were equally effective; however, there was less sleep latency and better sleep quality with 5 mg. No greater efficacy was attributed to doses higher than 5 mg.

Additionally, considering the eviswnce gathered in this review, higher doses could not be recommended as the most frequent side effects of melatonin including headache, dizziness, nausea or sleepiness, and as well as less frequent effects including shivering, anxiety, irritability, reduced alertness, and even hypotension must also be taken into consideration [49].

In this research study, doses were in the indicated range, and doses between 1 mg and 6 mg were shown to be effective in enhancing sleep in adults. Nonetheless, like Matheson et al. [48] pointed out, it was considered that further studies to find the optimal minimum effective dose are needed. The pharmacology and toxicology of melatonin requires a thorough systematic study based on well-designed clinical trials with sufficiently large population sizes that can extrapolate the results to the general population [5].

As indicated, failure to monitor endogenous melatonin levels is a problem for the validation of its supplements. Knowing the onset of lessened melatonin production in people is essential for early intervention [4].

On the other hand, it was considered that the participants’ diets should have been controlled since it has been shown that some nutritional factors. For instance, the intake of vegetables, caffeine (which was only controlled in two trials) and some vitamins and minerals can modify melatonin production, albeit to a lesser extent than light [50].

The indication related to alcohol abstinence is because alterations in the circadian secretion of melatonin and cortisol have been observed in alcoholics [51]. However, this issue has not been tested in healthy adults [52].

The outcomes of this review demonstrated a meaningful decrease in daytime sleepiness. Lemoine et al. [53] found no evidence of rebound effect in daytime sleepiness in the short or long term after treatment was stopped for 6 months.

In contrast, Shahrokhi et al. [54] confirmed an increase in daytime sleepiness after stopping treatment. Additionally, Besag et al. [55] reported that the most common adverse effect of exogenous melatonin administration was increased daytime sleepiness the following day. Nave et al. [56] suggested that melatonin administration before a short daytime nap might be associated with mild residual effects on psychomotor performance and significantly affect subjective feelings of sleepiness.

The review demonstrated a decrease in sleep onset latency in the intervention group. This outcome was supported by a meta-analysis that concluded that melatonin lessened sleep onset latency, increased total sleep time and improved overall sleep quality. The effects of melatonin on sleep are modest but do not appear to dissipate with continued melatonin use. Although the absolute benefit of melatonin compared with placebo is smaller than that of other pharmacological treatments for insomnia, melatonin may play a role in the treatment of insomnia due to its relatively benign side-effect profile in comparison with these agents [57].

Regardless basal endogenous melatonin levels, a meaningful reduction in sleep latency was demonstrated in an elderly population following exogenous melatonin administration. This effect remained or was even enhanced if the treatment was prolonged up to 6 months, with no signs of tolerance [58]. On the other hand, two other studies found no difference between exogenous melatonin administration and placebo regarding sleep latency [6,59].

Liu et al. [60] and Kuriyama et al. [47] also found positive effects of exogenous melatonin on sleep onset latency and sleep quality. However, in every case, these effects were small from a clinical point of view (weak recommendation for low quality evidence).

Another procured result of this review was the existence of few night-time awakenings. This conclusion is probably the most underpinned by the scientific literature. Several studies reached the same conclusion [61,62]. Eckerberg et al. [63] demonstrated that in young people, there was a meaningful decrease in night-time awakenings after one week of treatment with melatonin compared with placebo. Xie et al. [64] indicated that melatonin synchronized circadian rhythms and enhanced the onset, duration and quality of sleep.

An increase was found in the total night-time sleep period. It was shown that melatonin doses between 1 and 10 mg after the night shift might increase sleep time compared with placebo [6], and Wade et al. [58] demonstrated that although the overall half-life of melatonin products is approximately 30 to 60 min, having a soporific effect might influence sleep onset and maintenance.

MacFarlane et al. [65] reported that administration of high daily doses of melatonin for a couple of consecutive weeks resulted in a significant increase in total sleep time. These doses were higher than what was considered as hazardous [44]. According to Herxheimer et al. [5], there was no greater efficacy for melatonin doses higher than 5 mg.

Observations concerning daytime alertness in the melatonin-treated group were endorsed by Lemoine et al. [53], who found that in a population over 55 years of age, daily administration of 2 mg of prolonged-release melatonin for 3 weeks significantly enhanced sleep quality and daytime alertness compared to placebo administration. By the same token, MacFarlane et al. [65] demonstrated in their research the same evidence regarding the administration with high doses of melatonin.

Riha [66] concluded that one of the most frequent effects of melatonin administration was a decrease in alertness the following day, whereas Sharkey et al. [67] concluded that melatonin had no effect on alertness in shift workers. 

Lastly, there are reviews that indicated the effectiveness of melatonin in overcoming jet lag for occasional short-term use [5,68]; however, this was not the aim of this review.

### Limitations of the Review

The outcomes were limited by the few studies included in this review. Most of the studies did not specify whether they controlled for confounding factors that could affect the result. This reasserts the modest evidence and the level of recommendation obtained. 

Simultaneously, and as has been explained throughout this paper, important doubts were raised concerning the studied population (recruited professionals, number of participants and their age) and the non-mention of the melatonin type administered (fast- or slow-acting). 

The lack of homogeneity regarding population age (under 55 years), dose administered, interventions times and outcome measures hindered a meta-analysis that could support recommendations on the use of exogenous melatonin in shift workers. 

Another limitation was the few articles found and their lack of timeliness. This low number of papers found could imply that the search equation was very specific, raising doubt regarding a possible documentary silence. The manual search of the bibliographic lists of the included articles did not provide new documents to be included in the review without forgetting that the aim of this review was to ascertain the possible existence of evidence to recommend melatonin to health care professionals who usually work night shifts.

Certainly, some articles related to clinical practice may have been omitted from this review. Nonetheless, according to the Cochrane Collaboration, it was decided to include clinical trials in search of the maximum possible evidence.

## 5. Conclusions

Exogenously administered melatonin is effective in shift worker health personnel who are suffering from sleep disorders, and given its low adverse effects and tolerability, it might be recommended for this population. 

These findings are according to the clinical practice guidelines for the treatment of intrinsic circadian rhythm sleep–wake disorders [69] promoted by the American Academy of Sleep Medicine in its 2015 update, which bestowed positive approval to melatonin for the treatment of intrinsic circadian rhythm sleep–wake disorders with a second level of confidence.

A great disparity was evidenced terms of dose, follow-up periods, type of melatonin, small participant population, same age ranges and young age. Therefore, new trials would be needed to amend these observations to have full evidence that is able to ensure the efficacy of exogenous melatonin in the studied population.

## Figures and Tables

**Table 1:** Summary of accepted articles for review on the effects of exogenous melatonin on shift work-induced sleep disorder in health personnel.

Author, Year	Studied Population	Country	Intervention Period	Intervention Type	Observed Result
Marqueze et al., 2021 [16]	Population type: Nurses N total: 27 H/M: 0/27 Age: 37.1 ± 5.9 years	Brazil	24 weeks	Administration of 3 mg exogenous melatonin or placebo on nights when they were not working. Composite phase deviations (CPD) of mean sleep time based on actigraphy (efficiency and total sleep time) were calculated to measure circadian misalignment.	Significant 20% decrease in circadian misalignment (*p* < 0.001). As well as reduction in weight, waist and hip circumference.
Farahmand et al., 2018 [17]	Population type: emergency medicine residents N total: 24 (Gc:12-Gi: 12) H/M: 14/10 Age: 31.21 ± 5.23 years	Iran	4 weeks (From 19 May to 19 June 2016)	Take 3 mg melatonin, versus placebo, 1 h before bedtime for 2 consecutive days. The measurement was made using the Karolinska Sleep Scale	Melatonin therapy meaningfully lessened daytime sleepiness in comparison with placebo from the second night onwards (*p* = 0.003).
Sadeghniiat-Haghighi et al., 2016 [18]	Population type: Shift workers with difficulty to get to sleep N total: 50 (Gc: 25–Gi:25) H/M: Not recorded Age: Not recorded	Iran	3 nights’ treatment and 2 weeks washout period.	Take 3 mg melatonin, versus placebo, 30 min before bedtime. Total sleep time, sleep onset latency, sleep efficiency and awakening after sleep onset were analyzed.	Melatonin therapy improved sleep onset latency and decreased nocturnal awakenings, although there was no association when compared to the placebo group in relation to total sleep time and awakening after sleep onset (*p* > 0.05). Sleep onset latency and sleep efficiency improved significantly (*p* < 0.05).
Sadeghniiat-Haghighi et al., 2008 [19]	Population type: nurses with insomnia N total: 86 H/M: 0/86 Age: From 24 to 46 years	Iran	1 night treatment with melatonin and washing out for 4 days.	Oral intake of 5 mg melatonin taken 30 min before night-time sleep. Insomnia, subjective sleep onset latency, number of awakenings and sleep duration were measured.	While the subjects were taking melatonin (*p* < 0.05), sleep onset latency lessened meaningfully. There was no association when analysing the number of awakenings and sleep duration.
Cavallo et al., 2005 [20]	Population type: 2º year paediatric residents N total: 45 H/M: 16/29 Age: 28.6 ± 1.9 years	USA	2 Weeks	Taking melatonin (3 mg) vs. placebo before bedtime in the morning after the night shift. Standardized measures of sleep, mood and attention were assessed.	There were no significant differences in measures of sleep and mood. Significance was observed in the measure of attention (*p* = 0.03).
Yoon et al., 2002 [21]	Population type: Night shift nurses N total: 12 H/M: 0/12 Age: From 23 to 27 years	Korea	Follow-up for 9 days.	Three groups were set: placebo, melatonin, and melatonin with sunglasses. Melatonin (6 mg) was administered before bedtime for 2 days. Alertness, night-time sleep period and daytime sleep and mood were observed.	Total sleep period and total sleep times increased meaningfully with melatonin treatments (*p* < 0.05). Mood improved slightly. There was no significance between the melatonin treatment groups (with or without sunglasses).
Jockovich et al., 2000 [22]	Population type: emergency medicine residents N total: 19 H/M: 15/4 Age: 28.2 years	USA	3 consecutive days after each night shift.	Melatonin (1 mg) administration or placebo, 30 to 60 min before the daytime sleep session, for 3 consecutive days after each night shift. It was evaluated by Actigraph 1000 (efficiency and total sleep time). The mood profile and Stanford Sleepiness Scale were utilized to quantify mood and sleepiness.	There was no difference in sleep efficiency, duration, or latency (*p* > 0.05) between the melatonin group and placebo. Neither there was significance in mood profile and sleepiness (*p* > 0.05).
Wright et al., 1998 [23]	Population type: doctors N total: 15 H/M: 12/3 Age: From 32 to 45 years	USA	36 days (4 days for intervention, 28 days for washout and 4 days for intervention)	Melatonin (5 mg) administration or placebo for 3 consecutive nights after the night shift with crossover to the opposite agent after a subsequent block of night shifts. The primary outcome measure was the overall assessment of recovery as measured by a visual analogue scale. Secondary outcome measures included sleep quality, duration and fatigue. Furthermore, the Profile of Mood States and neuropsychological tests were used.	No beneficial effect of melatonin was found for sleep quality, fatigue or cognitive function in emergency physicians after the night shift (*p* < 0.05). The obtained results suggest that exogenous melatonin has limited value in the recovery of doctors after the night shift.
Jorgensen et al., 1998 [24]	Population type: resident doctors N total: 18 H/M: 16/2 Age: From 25 to 40 years	USA	5, 4, 3 and 2-night series	Administration of 10 mg sublingual melatonin or placebo every morning after the evening urgency. During daytime sleep periods, subjective sleep data were recorded. During night shifts, alertness was assessed using the Stanford Sleepiness Scale.	Melatonin improved daytime sleep and night-time alertness (*p* = 0.3); however, in neither case was the improvement statistically meaningful. Exogenous melatonin had a slight benefit in terms of improved alertness (*p* < 0.05).
James et al., 1998 [25]	Population type: night-shifts paramedics N total: 22 H/M: 17/5 Age: From 20 to 41 años	USA	A total of 4 consecutive night shifts (2 melatonin, 2 placebo)	Administration of melatonin 6 mg one capsule orally 30 min before each consecutive day’s sleep. Assessment of sleep quality, post-treatment mood and workload ratings were measured daily using a Visual Analogue Scale (VAS).	No clinical benefits were observed in staff working rotating night shifts. Melatonin was associated with meaningful fewer interim awakenings during daytime sleep compared with placebo (*p* < 0.05). For the rest of the studied variables, no significant differences were found (*p* > 0.05).

M/F = Rationship Male/Female; CG = Control Group; GI = Group Intervention.

**Table 2:** Evaluation of the adequacy of the studies through the 25 assessment items of the CONSORT guide.

	1	2	3	4	5	6	7	8	9	10	11	12	13	14	15	16	17	18	19	20	21	22	23	24	25	Total	%
Marqueze et al., 2021 [16]	0.5	1	1	1	1	1	1	1	1	0	0	1	1	1	0	1	1	1	0	1	1	1	1	1	1	20.5	13.71
Farahmand et al., 2018 [17]	1	1	1	1	1	0.5	0.5	1	0	0	0	0.5	1	0.5	1	1	1	1	0	1	1	1	0	1	1	18	12.04
Sadeghniiat-Haghighi et al., 2016 [18]	1	1	1	1	1	1	0	0	0	0	0	1	1	1	0	0	1	1	0	1	1	1	0	0	1	15	10.03
Sadeghniiat-Haghighi et al., 2008 [19]	1	1	0	1	1	0	0	0.5	0	0	0	0.5	0.5	0	1	1	1	1	1	1	1	1	0	0	1	14.5	9.69
Cavallo et al., 2005 [20]	0.5	1	1	1	1	1	1	0.5	0	1	0	1	1	0.5	1	1	1	1	1	1	1	1	0	0	1	19.5	13.04
Yoon et al., 2002 [21]	0.5	0.5	0.5	1	1	0.5	0	0	0	0	0	1	0.5	0.5	1	1	1	1	0	1	0	1	0	0	0	12	8.02
Jockovich et al., 2000 [22]	0.5	1	1	1	1	0.5	0.5	0	0	0	0	1	0.5	0.5	0	0	0	0	0	1	0	1	0	0	0	9.5	6.35
Wright et al., 1998 [23]	0.5	1	0.5	1	1	0.5	0	0	0	1	0.5	0.5	1	0.5	1	1	1	0	1	1	1	1	0	0	1	16	10.70
Jorgensen et al., 1998 [24]	0.5	0.5	1	1	1	0	0	0.5	0	0	1	0	0	0	0	0	0	0	0	1	1	0.5	0	0	1	9	6.02
James et al., 1998 [25]	1	1	1	1	1	1	1	0.5	0	1	0	0.5	1	0	0	1	0.5	0	1	1	1	1	0	0	0	15.5	10.36

**Table 3:** Study of the biases in the trials included in the review [13].

	1	2	3	4	5	6	7
Marqueze et al. [16]	No	No	Yes	Yes	No	No	No
Farahmand et al. [17]	No	No	No	Yes	Yes	No	No
Sadeghniiat-Haghighi et al. [18]	No	No	Yes	Yes	Yes	No	No
Sadeghniiat-Haghighi et al. [19]	No	No	No	No	Yes	No	No
Cavallo et al. [20]	No	No	No	No	Yes	Yes	Yes
Yoon et al. [21]	No	No	No	No	Yes	Yes	No
Jockovich et al. [22]	No	No	No	No	Unclear	Yes	Yes
Wright et al. [23]	No	Unclear	Unclear	Unclear	Yes	Yes	No
Jorgensen et al. [24]	No	No	No	No	Yes	No	Yes
James et al. [25]	No	No	Unclear	Yes	Yes	No	Yes
Selection bias (Generation of random sequences) Selection bias (Concealment of allocation) Performance bias (Blinding of participants and staff) Detection bias (Blinding of outcome assessment) Attrition bias (Unfinished result data) Reporting bias (Selective reporting) Other biases (Description of other sources of bias)							

## Data Availability

Not applicable.
